# Complete genome sequence of the *Phaeobacter gallaeciensis* type strain CIP 105210^T^ (= DSM 26640^T^ = BS107^T^)

**DOI:** 10.4056/sigs.5179110

**Published:** 2014-03-25

**Authors:** Oliver Frank, Silke Pradella, Manfred Rohde, Carmen Scheuner, Hans-Peter Klenk, Markus Göker, Jörn Petersen

**Affiliations:** 1 Leibniz Institute DSMZ – German Collection of Microorganisms and Cell Cultures, Braunschweig, Germany; 2 Helmholtz-Centre for Infection Research, Braunschweig, Germany

**Keywords:** *Alphaproteobacteria*, *Roseobacter* group, Plasmid wealth, Replication systems, Sister species, *Phaeobacter inhibens*

## Abstract

*Phaeobacter gallaeciensis* CIP 105210^T^ (= DSM 26640^T^ = BS107^T^) is the type strain of the species *Phaeobacter gallaeciensis*. The genus *Phaeobacter* belongs to the marine *Roseobacter* group (*Rhodobacteraceae, Alphaproteobacteria*). *Phaeobacter* species are effective colonizers of marine surfaces, including frequent associations with eukaryotes. Strain BS107^T^ was isolated from a rearing of the scallop *Pecten maximus*. Here we describe the features of this organism, together with the complete genome sequence, comprising eight circular replicons with a total of 4,448 genes. In addition to a high number of extrachromosomal replicons, the genome contains six genomic island and three putative prophage regions, as well as a hybrid between a plasmid and a circular phage. Phylogenomic analyses confirm previous results, which indicated that the originally reported *P. gallaeciensis* type-strain deposit DSM 17395 belongs to *P. inhibens* and that CIP 105210^T^ (= DSM 26640^T^) is the sole genome-sequenced representative of *P. gallaeciensis*.

## Introduction

Strain CIP 105210^T^ (= BS107^T^ = DSM 26640^T^) is the type strain of *Phaeobacter gallaeciensis*, the type species of *Phaeobacter*, a genus of marine species of *Rhodobacteraceae* (*Rhodobacterales*, *Alphaproteobacteria*). BS107^T^ was isolated from the scallop *Pecten maximus* and was initially described as the type strain of *Roseobacter gallaeciensis* [1]. After comprehensive reclassifications of *Rhodobacteraceae* genera, BS107^T^ became the type strain of the species *P. gallaeciensis* [2], currently comprising the species *P. gallaeciensis*, *P. inhibens, P. caeruleus*, *P. daeponensis*, *P. leonis* and *P. arcticus*. A recent study [3] revealed the non-identity of the reported identical deposits DSM 17395 and CIP 105210^T^ and confirmed that the strain CIP 105210^T^ represents the original *P. gallaeciensis* isolate BS107^T^, which is now deposited in the DSMZ open collection as DSM 26640^T^. In contrast, strain DSM 17395 was reclassified as a representative of the sister species *P. inhibens.* Analysis of their similar, but distinct metabolic capacities allowed for a discrimination between the two strains, which were originally reported to represent the same type strain [3]. Thus, in the absence of sequenced genomes, the assignment to species was essentially based on deviating plasmid profiles and molecular analyses (16S rDNA, ITS, DNA-DNA hybridization), which showed convergent results.

The genus *Phaeobacer* comprises effective surface colonizers. Comparative analyses of strains DSM 17395 and DSM 24588 (= 2.10) revealed a high level of adaptation to life on surfaces [4]. The production of the characteristic antibiotic tropodithietic acid (TDA) correlates with the formation of a brown pigment that is eponymous for *Phaeobacter* [1]. Current scientific interest in *Phaeobacter* is based on the role of its strains as probiotic agents in fish aquaculture [5] and as agents of bleaching diseases in marine red algae [6], as well as on their potential regulatory activity during phytoplankton blooms [7] *via* so-called roseobacticides [8]. Here we present the complete genome sequence of *P. gallaeciensis* CIP 105210^T^, together with a summary classification and a set of features, including insights into genome architecture, genomic islands and phages.

## Classification and features

### 16S rRNA gene analysis

**Figure 1:**
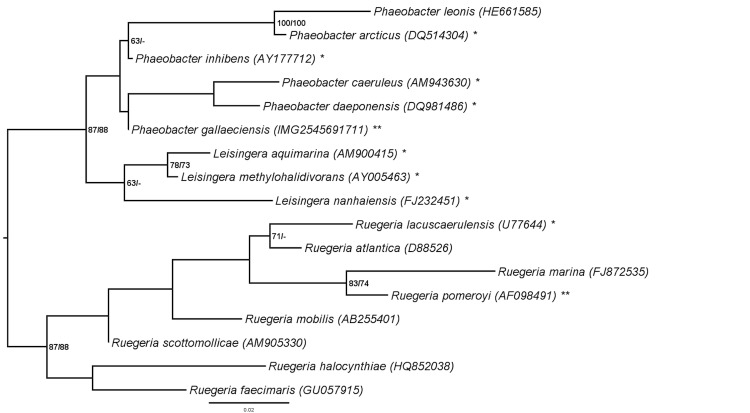
Phylogenetic tree highlighting the position of *P. gallaeciensis* relative to the type strains of the other species within the genus *Phaeobacter* and the neighboring genus *Leisingera*. The tree was inferred from 1,381 aligned characters of the 16S rRNA gene sequence under the maximum likelihood (ML) criterion as previously described [9]. *Ruegeria* spp. were included in the dataset for use as outgroup taxa. The branches are scaled in terms of the expected number of substitutions per site. Numbers adjacent to the branches are support values from 1,000 ML bootstrap replicates (left) and from 1,000 maximum-parsimony bootstrap replicates (right) if larger than 60% [9]. Lineages with type strain genome sequencing projects registered in GOLD [10] are labeled with one asterisk, those also listed as 'Complete and Published' with two asterisks. Genome sequences are available for *P. arcticus* (DQ514304) [11], *P. inhibens* (AY177712) [12], *P. caeruleus* (AM943630) [13], *P. daeponensis* (DQ981416) [14], *P. gallaeciensis* (IMG2545691711, this publication), *L. aquimarina* (AM900415) [15], *L. methylohalidivorans* (AY005463) [16] and *R. pomeroyi* (AF098491) [17].

Figure 1 shows the phylogenetic neighborhood of *P. gallaeciensis* CIP 105210^T^ in a 16S rDNA gene sequence based tree. The sequences of the four 16S rRNA identical gene copies in the genome differ by five nucleotides from the previously published 16S rDNA gene sequence (Y13244 [1]).

A representative genomic 16S rDNA gene sequence of *P. gallaeciensis* CIP 105210^T^ was compared with the Greengenes database for determining the weighted relative frequencies of taxa and (truncated) keywords as previously described [9], to infer the taxonomic and environmental affiliation of the strain. The most frequently occurring genera were *Ruegeria* (30.2%), *Phaeobacter* (29.4%), *Roseobacter* (13.9%), *Silicibacter* (13.7%) and *Nautella* (3.6%) (698 hits in total). Regarding the 30 hits to sequences from members of the species, the average identity within HSPs (high-scoring segment pairs) was 99.6%, whereas the average coverage by HSPs was 18.7%. Regarding the 20 hits to sequences from other members of the genus, the average identity within HSPs was 98.0%, whereas the average coverage by HSPs was 18.7%. Among all other species, the one yielding the highest score was *P. inhibens* (AY177712), which corresponded to a 16S rDNA gene identity of 99.5% and an HSP coverage of 18.6%. (Note that the Greengenes database uses the INSDC (= EMBL/NCBI/DDBJ) annotation, which is not an authoritative source for nomenclature or classification.) The highest-scoring environmental sequence was AJ296158 (Greengenes short name 'Spain:Galicia isolate str. PP-154'), which showed an identity of 99.8% and an HSP coverage of 18.7%. The most frequently occurring keywords within the labels of all environmental samples which yielded hits were 'microbi' (2.8%), 'marin' (2.5%), 'coral' (2.4%), 'sediment' (2.0%) and 'biofilm' (1.9%) (509 hits in total). Environmental samples which yielded hits of a higher score than the highest scoring species were not found.

### Morphology and physiology

**Figure 2:**
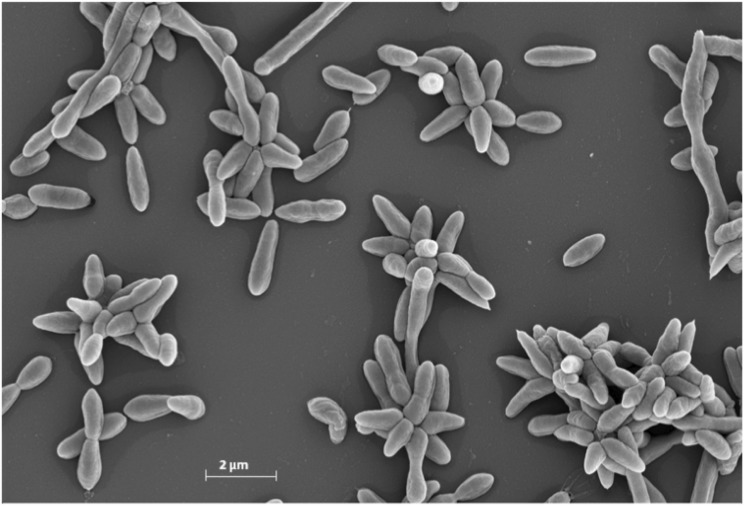
Scanning electron micrograph of P. gallaeciensis CIP 105210T.

Cells of BS 107^T^ stain Gram-negative and are ovoid-shaped rods ranging 0.7-1.0 µm in width and 1.7-2.5 µm in length. Motility is achieved by means of a polar flagellum (not visible in Figure 2). Young colonies grown on Marine Broth (MB) at 23°C are 0.5 mm in diameter, circular, smooth, convex and brownish with regular edges [1]. Colonies incubated for 7 days are 2 mm in diameter with irregular edges and produce a brown, diffusible pigment. Cells grow at temperatures between 15 and 37°C; optimal growth was observed in a range between 23 and 27°C. The optimal pH is 7.0, with growth occurring up to pH 10.0 but none below pH 4.0. Cells grow at salt concentrations ranging from 0.1 to 2.0 M NaCl, with 0.2 M being the optimal concentration. Additional thiamine (vitamin B_2_) is required for growth in minimal medium. Cells exhibit catalase and oxidase activity, but they do not exhibit amylase, gelatinase, ß-galactosidase, tweenase, DNase, urease, arginine dihydrolase, lysine decarboxylase and ornithine decarboxylase activities [1].

BS107^T^ is able to use the following substrates as sole carbon source and energy source: D- mannose, D-galactose, D-fructose, D-glucose, D-xylose, melibiose, trehalose, maltose, cellobiose, sucrose, meso-erythritol, D-mannitol, glycerol, D-sorbitol, meso-inositol, succinate, propionate, butyrate, γ-aminobutyrate, DL-hydroxybutyrate, 2-ketoglutarate, pyruvate, fumarate, glycine, L-*a*-alanine, p-alanine, L-glutamate, L-lysine, L-arginine, L-ornithine, L-proline, acetate and leucine. Bacteriochlorophyll *a* was not detected [1].

The metabolic properties of *Phaeobacter gallaeciensis* CIP 105210^T^ and the *P. inhibens* strains DSM 17395, DSM 24588 (= 2.10) and DSM 16374^T^ (= T5^T^) were compared using the more sensitive Phenotype MicroArray (PM) technology [3]. Here, using the statistical analysis (clustering and discretization) approaches as implemented in *“opm”* [18,19], the non-identity of strains CIP 105210^T^ and DSM 17395 could be demonstrated despite an overall similar physiology. Differences could be found regarding the respiration of tyramine, which was positive in DSM 17395 and negative in CIP 105210^T^, and for butyrate, for which respiration was found to be negative in DSM 17395 and positive in CIP 105210^T^ [3]. A summary of the classification and features of CIP 105210^T^ is presented in Table 1.

**Table 1:** Classification and general features of *P. gallaeciensis* BS107^T^ according to the MIGS recommendations [20] published by the Genome Standards Consortium [21].

**MIGS ID**	**Property**	**Term**	**Evidence code**
		Domain *Bacteria*	TAS [22]
		Phylum *Proteobacteria*	TAS [23]
		Class *Alphaproteobacteria*	TAS [24,25]
	Current classification	Order *Rhodobacterales*	TAS [25,26]
		Family *Rhodobacteraceae*	TAS [25,27]
		Genus *Phaeobacter*	TAS [1,28]
		Species *Phaeobacter gallaeciensis*	TAS [1]
	Subspecific genetic lineage (strain)	BS107^T^	TAS [1]
MIGS-12	Reference for biomaterial	Ruiz-Ponte et al. 1998	TAS [1]
	Gram stain	Gram-negative	TAS [1]
	Cell shape	ovoid-rod-shaped	TAS [1]
	Motility	motile, *via* polar flagella	TAS [1]
	Sporulation	not reported	
	Temperature range	15-37°C, mesophile	TAS [1]
	Optimum temperature	23-27°C	TAS [1]
	Salinity	0.1-2.0 M NaC1	TAS [1]
MIGS-22	Relationship to oxygen	aerobe	TAS [1]
	Carbon source	complex substrates, butyrate, DL-hydroxybutyrate, D-xylose	TAS [1]
	Energy metabolism	chemoheterotrophic	TAS [1]
MIGS-6	Habitat	seawater, *Pecten maximus*	TAS [1]
MIGS-6.2	pH	4.0-10.0, optimum 7.0	TAS [1]
MIGS-15	Biotic relationship	free living, facultative symbiont	TAS [1]
MIGS-14	Known pathogenicity	-	IDA
MIGS-16	Specific host	*Pecten maximus*	
MIGS-18	Health status of host	not reported	
	Biosafety level	1	TAS [29]
MIGS-19	Trophic level	heterotroph	TAS [1]
MIGS-23.1	Isolation	seawater of larval cultures of the scallop *Pecten maximus*	TAS [1]
MIGS-4	Geographic location	A Coruna, Galicia, Spain	TAS [1]
MIGS-5	Time of sample collection	not reported	
MIGS-4.1	Latitude	43.3619	
MIGS-4.2	Longitude	-8.410	
MIGS-4.3	Depth	not reported	
MIGS-4.4	Altitude	about sea level	

Evidence codes - IDA: Inferred from Direct Assay; TAS: Traceable Author Statement (i.e., a direct report exists in the literature); NAS: Non-traceable Author Statement (i.e., not directly observed for the living, isolated sample, but based on a generally accepted property for the species, or anecdotal evidence). Evidence codes are from of the Gene Ontology project [30].

### Chemotaxonomy

The chemical composition of strain BS107^T^ confirmed ubiquinones as the sole respiratory lipoquinones and revealed Q10 as predominant. Polar lipids consisted of an unidentified phospholipid, two uncharacterized lipids, aminolipids, phosphatidylenthanolamine, phosphatidylglycerole and phosphatidylcholine [2].

The major fatty acids are the monounsaturated acids C_18:1 ω7c_ (76.1%), and 11-methyl C_18:1 ω7c_ (6.1%), followed by hydroxy fatty acid C_16:0 2-OH_ (5.1%) as well as C_16:0_ (4.0%), C_14:1_ (3.1%), C_18:0_ (2.6%), C_10:0 3-OH_ (2.2%) and C_18:1 ω9c_ (0.9%) [2].

## Genome sequencing and annotation

### Growth conditions and DNA extraction

A culture of CIP 105210^T^ was grown aerobically in 100 ml of DSMZ medium 514 [31] on a shaker at 28°C. Genomic DNA was isolated using the Qiagen Genomic DNA Kit, following the standard protocol for Bacteria 500G provided by the manufacturer. The extracted DNA had a concentration of 200 ng/µl. The quality of the DNA was checked with the NanoDrop.

### Genome sequencing and assembly

The genome of *P. gallaeciensis* CIP 105210^T^ was sequenced using the Roche/454 GS FLX Titanium sequencing platform [Table 2]. A draft assembly based on 247,768 reads of a standard shotgun library and 204,863 reads of a 3 kbp paired-end library (LGC Genomics, Berlin, Germany) with a total of 138 Mb (22-fold coverage) was generated with Newbler assembler, Roche Diagnostics GmbH, Mannheim, Germany). This assembly consisted of 45 contigs 26 of which could be joined into 15 scaffolds. Gaps resulting from repetitive sequences were closed by PCR followed by Sanger sequencing, yielding a final genome size of 4,540,155 bp, that consists of one circular chromosome of 3,776,653 bp and seven circular plasmids.

**Table 2:** Genome sequencing project information

MIGS ID	Property	Term
MIGS-31	Finishing quality	Finished
MIGS-28	Libraries used	One draft assembly of standard shotgun library, one 3 kbp paired-end library
MIGS-29	Sequencing platforms	Roche/454 GS FLX Titanium
MIGS-31.2	Sequencing coverage	22 ×
MIGS-30	Assemblers	Newbler assembler version 2.6 (Software Release: 2.6 (20110517_1502)
MIGS-32	Gene calling method	Prodigal 1.4
	INSDC ID	Pending
	GenBank Date of Release	Pending
	GOLD ID	Gi24053
	NCBI project ID	188096
	Database: IMG	2531839720
MIGS-13	Source material identifier	CIP 105210^T^
	Project relevance	Tree of Life, carbon cycle, scallop rearing, plasmid

### Genome annotation

Genes were identified using Prodigal [32] as part of the Integrated Microbial Genomes Expert Review (IMG/ER) annotation pipeline [33]. The predicted CDSs were translated and used to search the National Center for Biotechnology Information (NCBI) nonredundant database, UniProt, TIGR-Fam, Pfam, PRIAM, KEGG, COG, and InterPro databases.

## Genome properties

The *Phaeobacter gallaeciensis* CIP 105210^T^ genome statistics are provided in Table 3 and Figures 3a, 3b, 3c, 3d, 3e, 3f, 3g, 3h. The genome consists of eight circular replicons with a total length of 4,540,155 bp and a G+C content of 59.44%. The replicons correspond to a single chromosome (3,776,653 bp) and seven extrachromosomal elements ranging in size between 255,493 bp and 40,170 bp. From a total of 4,448 predicted genes, 4,369 were protein coding genes and 79 RNA genes. The distribution of genes into COGs functional categories is presented in Table 4.

**Table 3:** Genome statistics

Attribute	Value	% of Total
Genome size (bp)	4,540,155	100.00
DNA coding region (bp)	4,056,108	89.34
DNA G+C content (bp)	2,698,552	59.44
Number of replicons	8	
Extrachromosomal elements	7	
Total genes	4,448	100.00
RNA genes	79	1.78
rRNA operons	4	
tRNA genes	59	1.33
Protein-coding genes	4,369	98.22
Genes with function prediction	3,595	80.82
Genes in paralog clusters	3,475	78.13
Genes assigned to COGs	3,422	76.93
Genes assigned Pfam domains	3,657	82.22
Genes with signal peptides	457	10.27
Genes with transmembrane helices	975	21.92
CRISPR repeats	0	

**Figure 3a:**
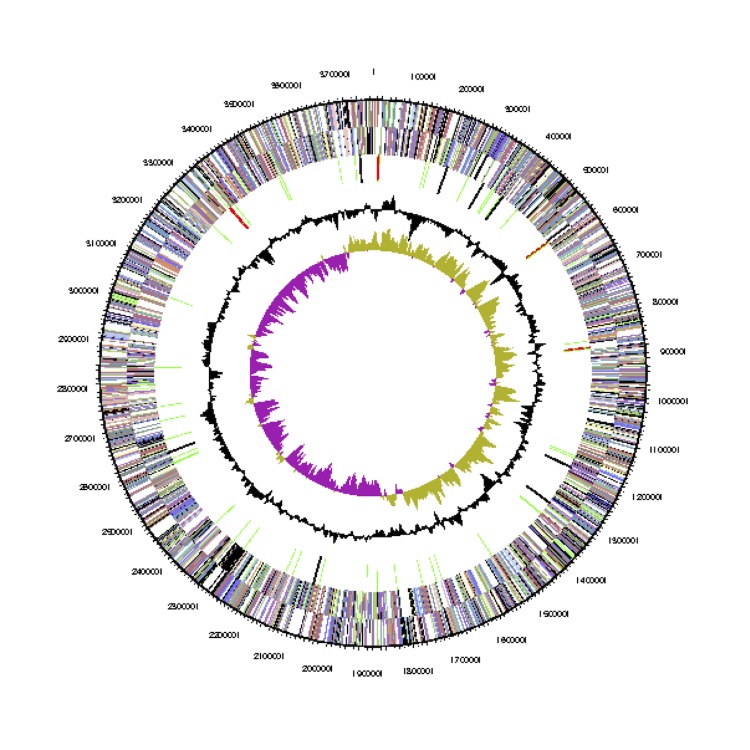
Circular graphical map of the chromosome. From margin to center: Genes on forward strand (colored by COG categories), genes on reverse strand (colored by COG categories), RNA genes (tRNAs green, rRNAs red, other RNAs black), GC content, GC skew.

**Figure 3b:**
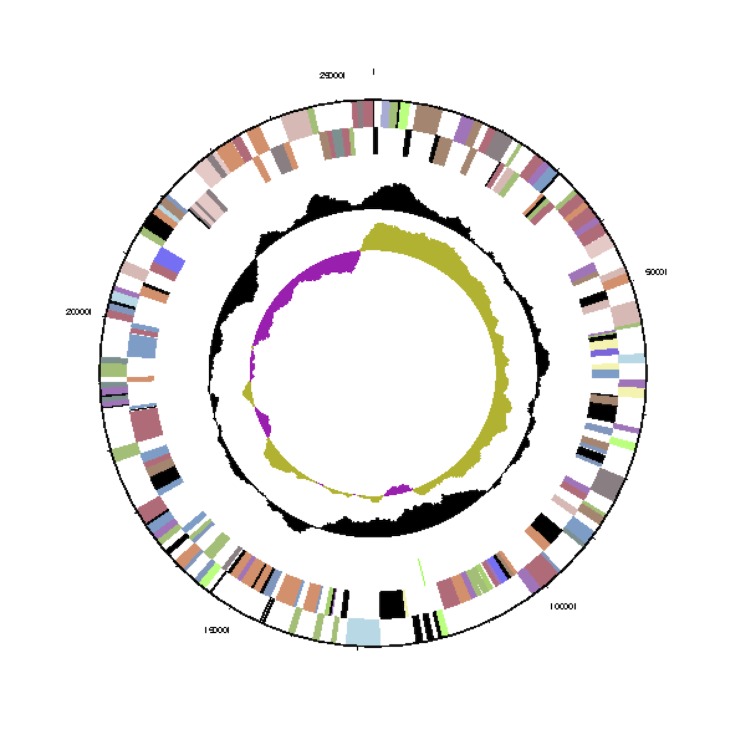
Circular graphical map of the extrachromosomal replicon pGal_A255. From margin to center: Genes on forward strand (colored by COG categories), genes on reverse strand (colored by COG categories), RNA genes (tRNAs green, rRNAs red, other RNAs black), GC content, GC skew.

**Figure 3c:**
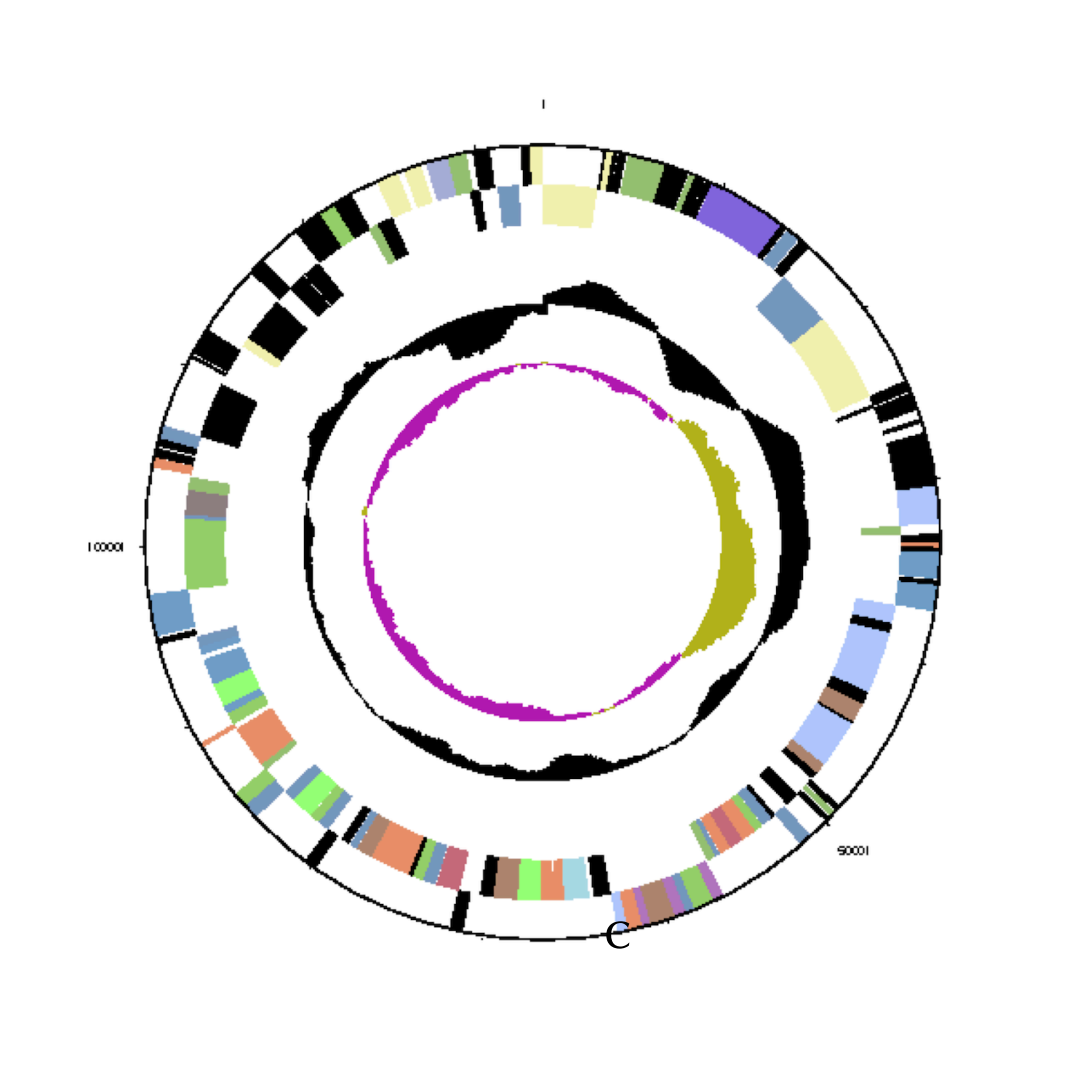
Circular graphical map of the extrachromosomal replicon pGal_B134. From margin to center: Genes on forward strand (colored by COG categories), genes on reverse strand (color by COG categories), RNA genes (tRNAs green, rRNAs red, other RNAs black), GC content, GC skew.

**Figure 3d:**
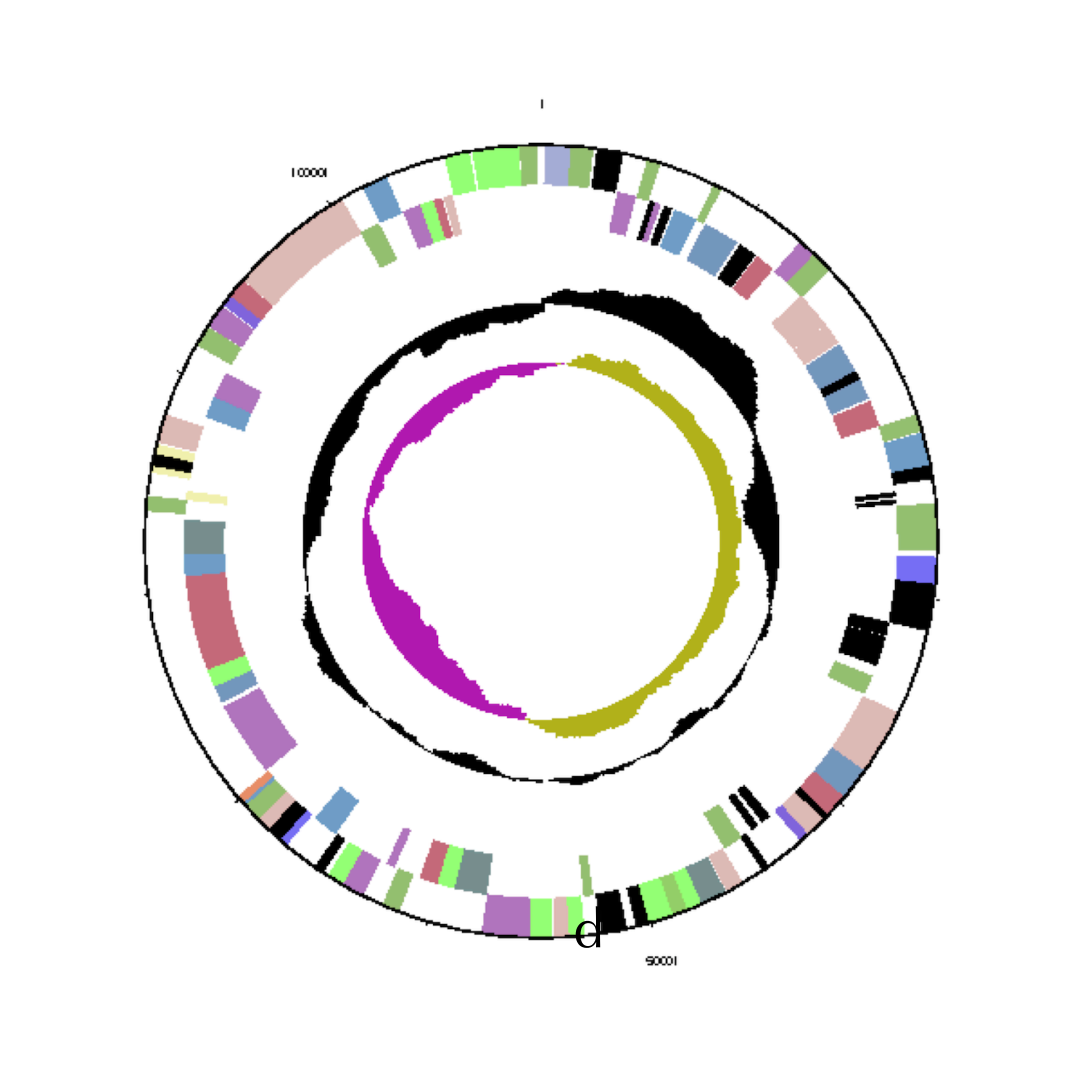
Circular graphical map of the extrachromosomal replicon pGal_C110. From margin to center: Genes on forward strand (colored by COG categories), genes on reverse strand (colored by COG categories), RNA genes (tRNAs green, rRNAs red, other RNAs black), GC content, GC skew.

**Figure 3e:**
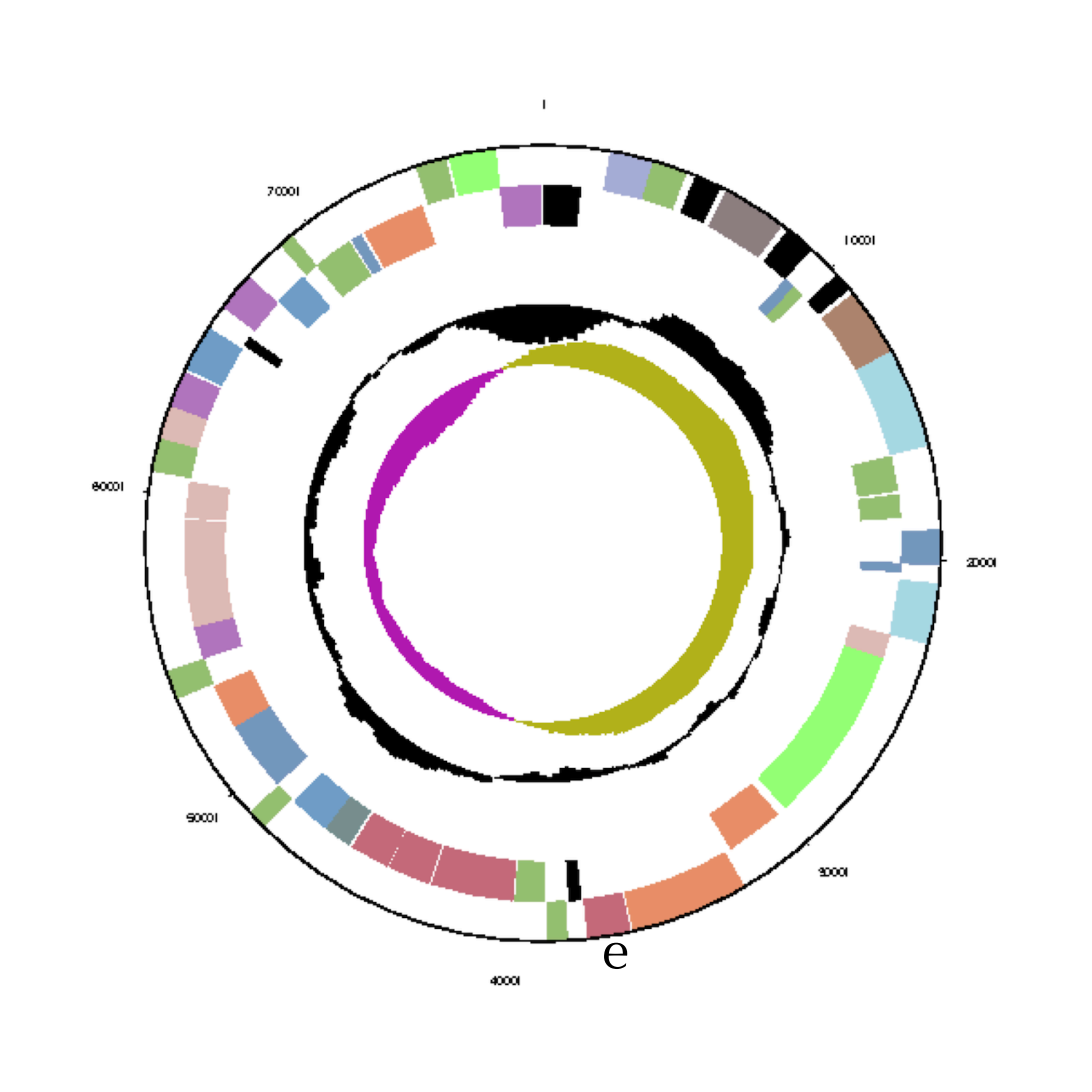
Circular graphical map of the extrachromosomal replicon pGal_D78. From margin to center: Genes on forward strand (colored by COG categories), genes on reverse strand (color by COG categories), RNA genes (tRNAs green, rRNAs red, other RNAs black), GC content, GC skew.

**Figure 3f:**
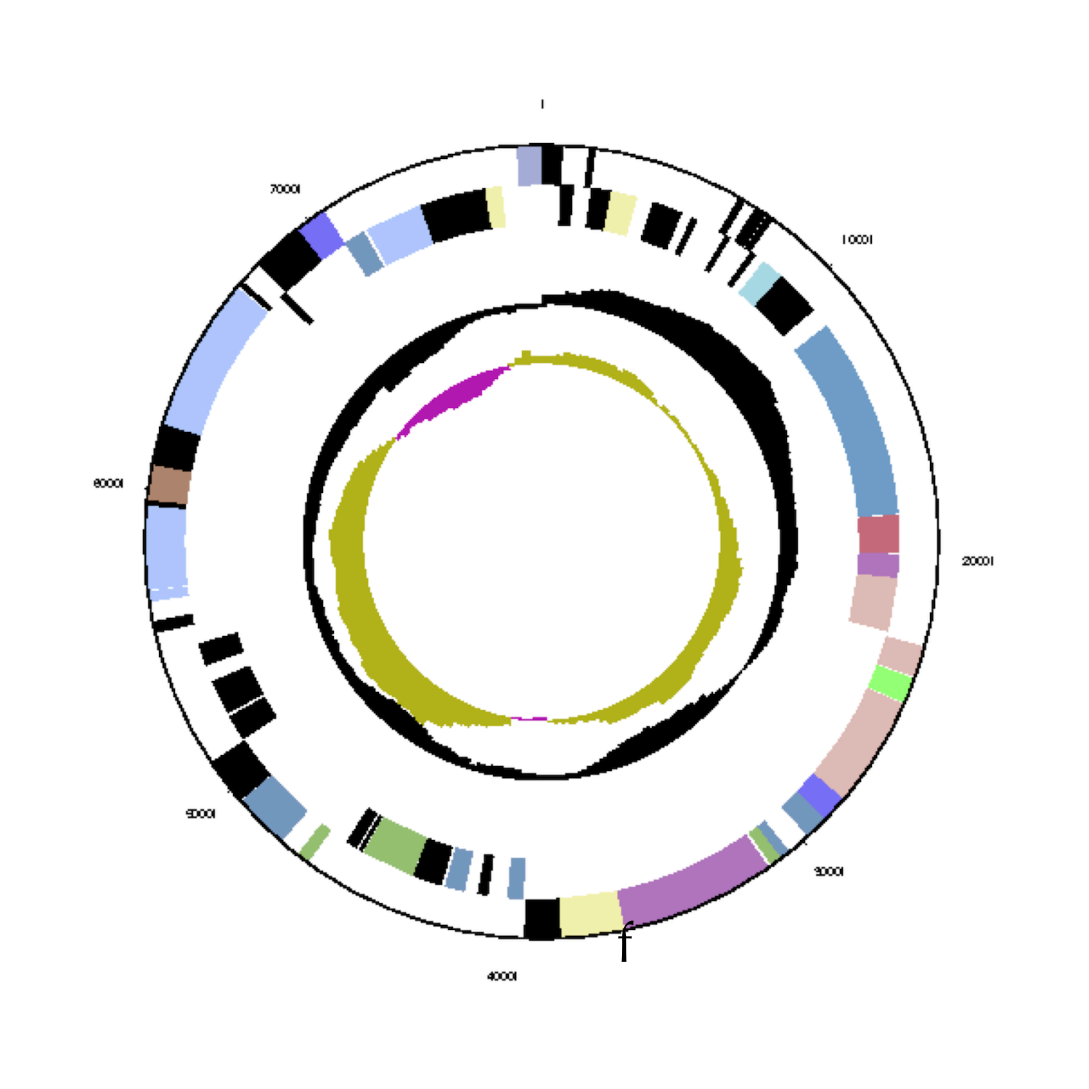
Circular graphical map of the extrachromosomal replicon pGal_E78. From margin to center: Genes on forward strand (colored by COG categories), genes on reverse strand (colored by COG categories), RNA genes (tRNAs green, rRNAs red, other RNAs black), GC content, GC skew.

**Figure 3g:**
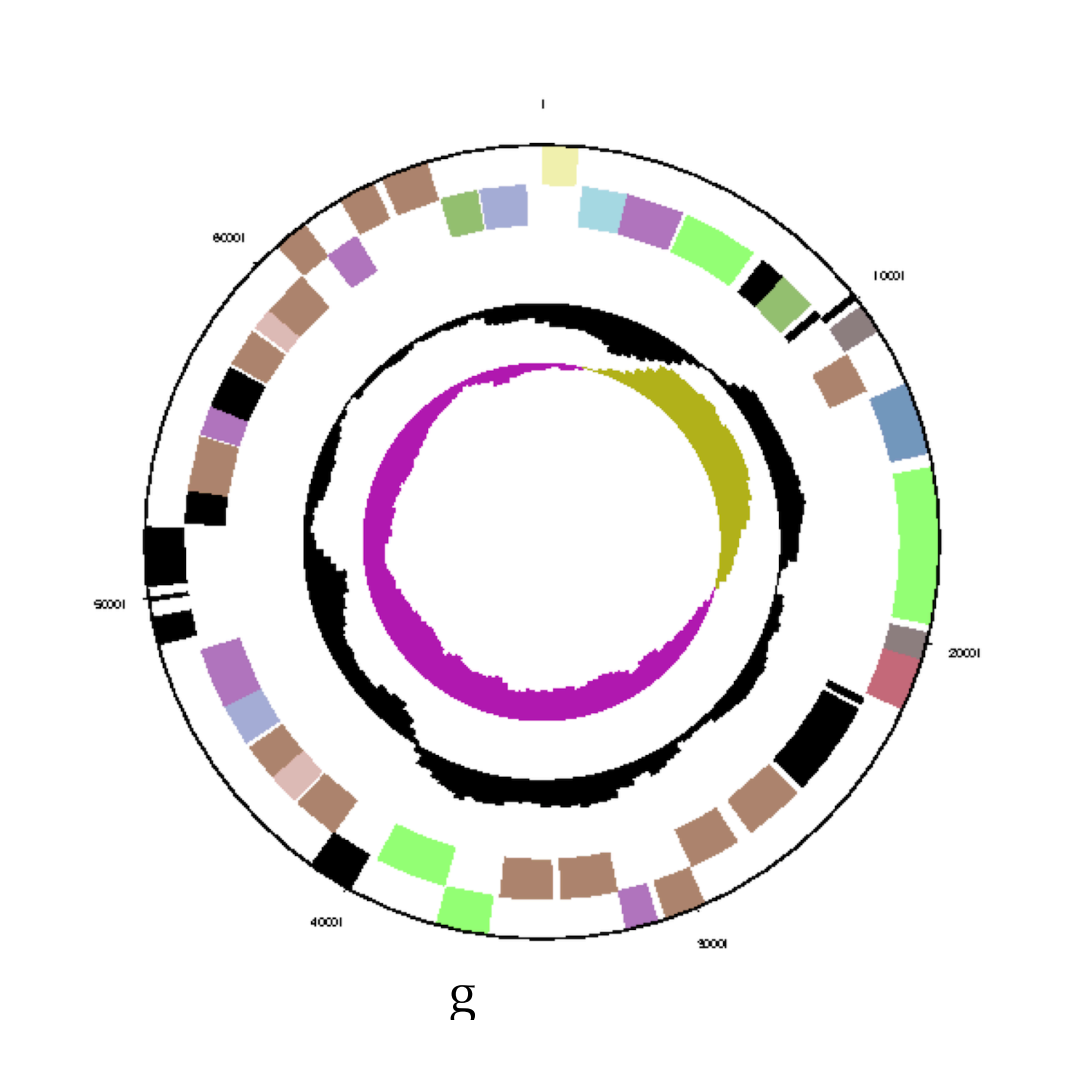
Circular graphical map of the extrachromosomal replicon pGal_F69. From margin to center: Genes on forward strand (colored by COG categories), genes on reverse strand (colored by COG categories), RNA genes (tRNAs green, rRNAs red, other RNAs black), GC content, GC skew.

**Figure 3h:**
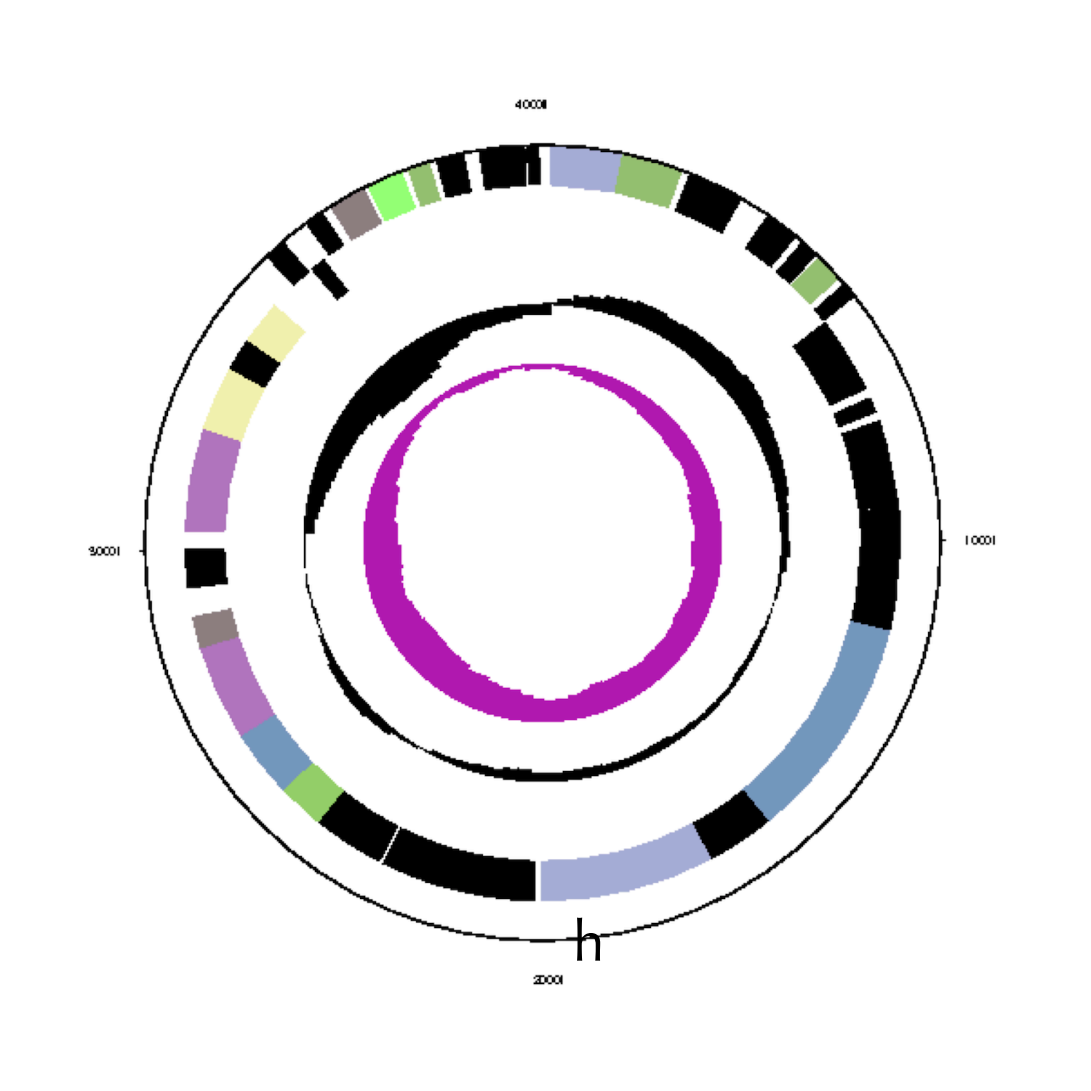
Circular graphical map of the extrachromosomal replicon pGal_G40. From margin to center: Genes on forward strand (colored by COG categories), genes on reverse strand (colored by COG categories), RNA genes (tRNAs green, rRNAs red, other RNAs black), GC content, GC skew.

**Table 4:** Number of genes associated with the general COG functional categories

**Code**	**Value**	**%age**	**Description**
J	170	4.51	Translation, ribosomal structure and biogenesis
A	1	0.03	RNA processing and modification
K	310	8.23	Transcription
L	150	3.98	Replication, recombination and repair
B	3	0.08	Chromatin structure and dynamics
D	35	0.93	Cell cycle control, cell division, chromosome partitioning
Y	0	0	Nuclear structure
V	50	1.33	Defense mechanisms
T	163	4.33	Signal transduction mechanisms
M	204	5.41	Cell wall/membrane/envelope biogenesis
N	53	1.41	Cell motility
Z	0	0.00	Cytoskeleton
W	0	0	Extracellular structures
U	87	2.31	Intracellular trafficking, secretion, and vesicular transport
O	146	3.87	Posttranslational modification, protein turnover, chaperones
C	235	6.24	Energy production and conversion
G	212	5.63	Carbohydrate transport and metabolism
E	436	11.57	Amino acid transport and metabolism
F	81	2.15	Nucleotide transport and metabolism
H	160	4.28	Coenzyme transport and metabolism
I	151	4.01	Lipid transport and metabolism
P	205	5.44	Inorganic ion transport and metabolism
Q	132	3.50	Secondary metabolites biosynthesis, transport and catabolism
R	451	12.00	General function prediction only
S	333	9.00	Function unknown
-	1,026	23.07	Not in COGs

## Insights into the genome

### Unique genes

A search for specific genes in the genome of *P. gallaeciensis* CIP 105210^T^ compared to the *P. inhibens* strains DSM 24588 (= 2.10), DSM 16374^T^ (= T5^T^) and DSM 17395, based on an e-value of 1e-5 and a minimum identity of 30%, resulted in a total number of 551 specific genes. 296 (54%) of these genes were located on the chromosome and 255 (46%) on extrachromosomal replicons. In comparison with the other completely sequenced bacterial strains of the genus *Phaeobacter*, 8% of the chromosomal and 35% of the extrachromosomal *P. gallaeciensis* CIP 105210^T^ genes were unique, thus reflecting the considerable contribution of extrachromosomal elements to unique gene content.

The observed distribution may be influenced by the presence of two chromosome-encoded bacterial MobC mobilization proteins (Gal_00154, Gal_01073). MobC, which is missing in all three completely sequenced *P. inhibens* strains, is part of the relaxosome at the origin of transfer and increases the frequency of plasmid mobilization and therefore conjugal transfer of plasmids [34], which is also in agreement with the comparably large number of seven extrachromosomal replicons present in CIP 105210^T^.

The probable function of some of the unique genes is explained below. Genes Gal_01405 and Gal_01407 constitute methane monooxygenases (EC 1.14.13.25) facilitating the degradation of aromatic compounds and phenols [35]. Gal_01397, a monoamine oxidase could provide an additional source of ammonium [36].

Unique genes are also provided by phage-like elements. In CIP 105210^T^ these so-called “morons” (because they add “more on” the genome [37]) comprise, e.g., an ABC-2 family drug transporter (Gal_01752) [38], and a negative regulator of beta-lactamase expression (Gal_02239).

### Genomic islands

Six genomic islands could be identified on the chromosome with the web-based island-viewer system [39]. Island-viewer combines the methods IslandPick [40], which uses a comparative genomics approach, SIGI-HMM [41], which relies upon deviating codon usage signatures, and IslandPath-DIMOB [42], which identifies genomic islands based on deviating GC content, dinucleotide bias in gene clusters and the presence of island specific genes like mobility genes and tRNAs.

Island-I ranging from position 155,977 to 177,667 (21,690 bp) contains a tRNA gene (Phe GAA, Gal_00137) next to a site-specific recombinase XerD (Gal_00138) and the bacterial mobilization protein (MobC, Gal_00154; see above). Furthermore, it contains a transcriptional regulator of the LysR family (Gal_00160) and an adjacent ABC-type transport system for glycine/proline/betaine. Island-II (422,441 to 434,165; 11,725 bp) mainly consists of hypothetical proteins, but it also contains a large type II restriction enzyme (905aa, Gal_00442) and another site specific XerD recombinase (Gal_00444) next to a tRNA for proline (Gal_00445). Island-III (1,085,143 to 1,096,105; 10,962 bp) contains three XerD recombinases in row (Gal_01065 to Gal_01067, a MobC protein (Gal_01073) and the typical VirD2 relaxase (Gal_01074) as well as the VirD4 coupling protein (Gal_01075) of type IV secretion systems [43] indicating a plasmid-derived origin of this island. Island-IV (1,626,663 to 1,641,677; 15,014 bp) contains an ABC-type cobalt transport system and a XerC recombinase (Gal_01616). Island-V (2,821,359 to 2,848,860; 27,501 bp) consists mainly of regulated TRAP C4-dicarboxylate and ABC-type dipeptide/oligopeptide/nickel transport proteins and also the epsilon subunit of DNA polymerase III (Gal_02817). Island-VI (3,328,870 to 3,344,910; 16,040 bp) lies adjacent to a ribosomal rRNA-operon and contains an ABC-type amino acid/amide transport system and an E1 component of the pyruvate dehydrogenase complex (Gal_03286, E.C.: 1.2.4.1).

### Phage-like elements

The presence of phage-like elements was analyzed with the online tool PHAST [44]. The program identified 16 genes representing a gene transfer agent (GTA [45];) and three incomplete clusters of phage-derived genes with sizes between 15 kb and 40 kb (Table 5).

**Table 5:** Prophage regions in the chromosome of *P. gallaeciensis* CIP 105210^T†^

Region	Length	Completeness	Score	CDS	Coordinates	Specific keyword	GC%
1	14.2 kb	Questionable	80	16	1,566,488-1,580,693	Gene transfer agent (GTA)	64.6
2	25.1 kb	Incomplete	30	32	1,781,279-1,806,383	integrase, region invertase, helicase	56.9
3	14.7 kb	Incomplete	40	18	1,800,767-1,815,474	Portal protein, head maturation protease	57.7
4	39.6 kb	Incomplete	60	45	2,265,763-2,305,412	Integrase, peptidoglycan hydrolase	58.5

^†^Completedness, a prediction of whether the region contains an intact or incomplete prophage based on the applied criteria of PHAST; Score, the score of the region based on the applied criteria of PHAST; CDS, the number of coding sequences; Coordinates, the start and end positions of the region on the bacterial chromosome; GC%, the percentage of GC nucleotides of the region.

### Extrachromosomal replicons

Complete genome sequencing of *Phaeobacter gallaeciensis* CIP 105210^T^ resulted in eight replicons ranging from 40 kb to 3.8 MB in size. For the seven extrachromosomal replicons, ranging in size between 40 kb and 255 kb (Table 6), circular confirmation has been experimentally validated. The extrachromosomal replicons were analyzed as described in [46] and [47]. They contain characteristic replication modules [43] of the RepABC-, DnaA-like, RepA- and RepB-type comprising a replicase and a parAB partitioning operon [48]. Plasmid pGal_E78 also contains a replicase that is homologous to those of RepABC-type plasmids, but the partitioning genes repAB are missing. The solitary replicase cannot be classified according to the established scheme [49] and is designated as RepC_soli-1a (RepC' [50]). The respective replicases of the other extrachromosomal replicons that mediate the initiation of replication are designated according to the established classification scheme [51]. The numbering of specific replicases corresponds to plasmid compatibility groups that are required for a stable coexistence of the replicons within the same cell [49].

**Table 6:** General genomic features of the chromosome and extrachromosomal elements from *P. gallaeciensis* strain CIP 105210^T#^

**Replicon**	**No.**	**Replicase**	**Length** (bp)	**GC** (%)	**Topology**	**No. Genes^#^**
Chromosome	1	DnaA	3,776,653	60	circular	3,703
pGal_A255	2	DnaA-like-I	255,493	58	circular	237
pGal_B134	3	RepABC-5	133,631	60	circular	155
pGal_C110	4	RepABC-8	109,815	56	circular	115
pGal_D78	5	RepB-I	77,876	62	circular	62
pGal_E78	6	RepC_soli-1a	77,775	55	circular	81
pGal_F69	7	RepA-I	68,752	58	circular	56
pGal_G40	8	RepABC-4	40,170	56	circular	51

^#^deduced from automatic annotation.

The comparison of the extrachromosomal replicons from *P. gallaeciensis* CIP 105210^T^ and *P. inhibens* DSM 17395 documents a strong conservation and long-range synteny of three replicons. The largest 255 kb DnaA-like-I replicon (pGal_A255) is slightly smaller than the 262 kb equivalent (NC_018291.1), sharing 89% identity on nucleotide level. The RepB-I type replicon pGal_D78 exactly matches the size of the DSM 17395 replicon (NC_018287.1, 91% identity), whereas the RepA-I type replicon pGal_F69 is slightly larger than its equivalent (65 kb; NC_018288.1, 91% identity). On the contrary, RepABC-type replicons are not present in the DSM 17395 genome. However, only two of the four additional plasmids, the RepABC-5 type replicon pGal_B134 and the RepC_soli-1a-type replicon pGal_E78 possess type IV secretion systems that are required for conjugative transfer [52]. Finally, the three replicons pGal_A255, pGal_B134, pGal_C110 are equipped with stabilizing toxin/antitoxin modules [53] (Table 7).

**Table 7:** Integrated Microbial Genome (IMG) locus tags of *P. gallaeciensis* CIP 105210^T^ genes for the initiation of replication, toxin/antitoxin modules and type IV secretion systems (T4SS) required for conjugation.

Replicon	Replication Initiation		Plasmid Stability		Type IV Secretion	
	Replicase	Locus Tag	Toxin	Antitoxin	VirB4	VirD4
Chromosome	DnaA	Gal_00001	-	-	-	-
pGal_A255	DnaA-like-I	Gal_03722	Gal_03770	Gal_03771	-	-
pGal_B134	RepABC-5	Gal_03960	Gal_03975	Gal_03974	Gal_04010	Gal_03992
pGal_C110	RepABC-8	Gal_04107	Gal_04110	Gal_04111	-	-
pGal_D78	RepB-I	Gal_04221	-	-	-	-
pGal_E78	RepC_soli-1a	Gal_04283	-	-	Gal_04360	Gal_04345
pGal_F69	RepA-I	Gal_04364	-	-	-	-
pGal_G40	RepABC-4	Gal_04417	-	-	*-*	-

**Figure 4:**
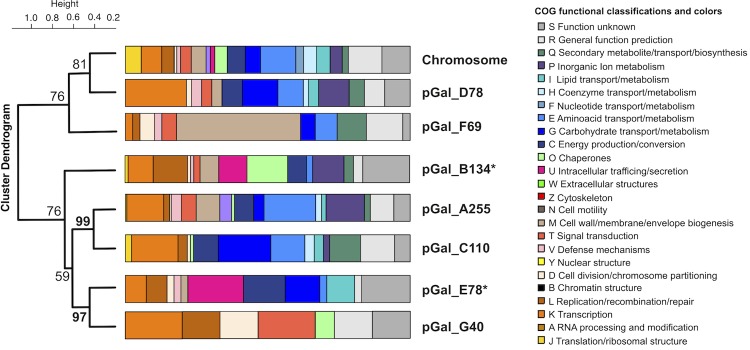
Summary and representation of the assortment of replicons in CIP 105210^T^ as previously described in [47]. Bars show the relative frequency of functional classes according to the database of clusters of orthologous groups of proteins (COGs). The cluster dendrogram arranges the replicons according to their overall codon usage. Codon-usage matrices were generated using yet unpublished scripts for the statistical analysis software R (version 2.15.0.) [54]. The hierarchical clustering analysis was conducted using the pvclust function [55] with “complete” as agglomeration method. Pvclust also returns the AU (Approximately Unbiased) values as statistical support for clusters in percent. Support values >95% are given in bold. Asterisks indicate the presence of genes for conjugation (T4SS) as listed in Table 7.

The 255 kb DnaA-like-I replicon pGal_A255 is largely constituted by genes coding for proteins in COG E “amino-acid transport and metabolism” and COG P “inorganic ion metabolism” (Figure 4). The latter category comprises, for example, a Fe3+ siderophore complex (Gal_03846 to Gal_03848), which contains ferric-iron chelating agents that facilitate enhanced uptake of this essential compound [56]. pGal_A255 furthermore harbors six genes involved in chemotaxis, a tRNA (Gal_03828) and a cluster for the biosynthesis of coenzyme PQQ, a redox factor (Gal_03896). The genes for the synthesis of the antibiotic tropodithietic acid (TDA) [57] are consolidated in a cluster on pGal_A255 and comprise *tdaA* (Gal_03819), *tdaB* (Gal_03818), *tdaC* (Gal_03817), *tdaE* (Gal_03815) and *tdaF* (Gal_03802). The 134 kb RepABC-5 type plasmid pGal_B134 harbors in comparison to the other seven replicons the most chaperons (COG O, Figure 4), owing to an elevated presence of cytochromes and disulfide bond formation proteins. pGal_B134 also holds a dimethyladenosinetransferase (Gal_03978) that facilitates RNA methylation and a T4S system (Table 7), thus combining on this plasmid genes for epigenetic modifications. The RepABC-8 type plasmid pGal_C110 consists mainly of amino acid and carbohydrate transporters (COGs E and G) and biogenesis of secondary metabolites (COG Q). COG K, transcription is also elevated, due to the presence of 15 transcriptional regulators. On the RepB-I replicon pGal_D78, COG K transcription is elevated, owing to the presence of twelve transcriptional regulators and a RNA-polymerase (Gal_04277). This replicon also contains genes for siderophore synthetases (Gal_04241 to Gal_04247) and a catalase/peroxidase (Gal_04279). On the RepC_soli-1a plasmid pGal_E78, proteins of COG C energy production and conversion are constituted by pyruvate dehydrogenase E1 and E2 components, which play a role in the citrate cycle and gluconeogenesis. The RepA-I replicon pGal_F69 contains an RTX toxin [58] (Gal_04412) and exhibits a strong accumulation of COG M, “cell-envelope biogenesis”. It harbors several polysaccharide export proteins including a type I secretion system ABC transporter (Gal_04381, Gal_04382), and a complete rhamnose operon [59]. *P. gallaeciensis* CIP 105210^T^ (= DSM 26440^T^) forms strong biofilms (unpublished results) and the extrachromosomal 69 kb replicon seems to be responsible for the attached lifestyle as previously proposed for the *P. inhibens* strains DSM 17395 and DSM 24588 (2.10) [3]. pGal_G40 represents a hybrid between a plasmid and a circular phage, comparable to the coliphage N15 [60,61]. It contains an N-acyl-L-homoserine lactone synthetase (Gal_04460) and a complete *repABC* operon. This interesting finding draws a direct connection between RepABC directed replication [49], horizontal gene transfer and AHL-mediated quorum sensing [62].

Genome sequencing of *P. inhibens* DSM 16374^T^ (T5^T^) revealed the presence of the complete dissimilatory nitrate reduction pathway and anaerobic growth on nitrite has been validated experimentally [12]. The genes of the pathway are located on three different replicons, i.e. the chromosome, the DnaA-like I type plasmid pInhi_A227 and the RepABC-8 type plasmid pInhi_B88. The genome of the sister species *P. gallaeciensis* CIP 105210^T^ exhibits a conspicuous synteny for the chromosome and three extrachromosomal replicons (DnaA-like I (pGal_A255, pInhi_A227), RepB-I (pGal_D78, pInhi_C78), RepA-I (pGal_F69, pInhi_D69)). However, the RepABC-8 type plasmid including the crucial nitrous oxide reductase (EC 1.7.2.4) is missing in *P. gallaeciensis* CIP 105210^T^, and this strain is accordingly unable to grow anaerobically.

### Phylogenomic analyses

The phylogenetic analysis of 16S rRNA gene type-strain sequences places *P. gallaeciensis* together with both *P. caeruleus* and *P. daeponensis*, whereas *P. inhibens* forms a cluster with *P. leonis* and *P. arcticus*. Both clusters are set apart from each other, but the 16S rRNA gene tree is unresolved and does not allow one to infer the evolutionary interrelationships in this group. Previous results [4] showed that the reported *P. gallaeciensis* type-strain deposit DSM 17395 belongs to *P. inhibens* and that CIP 105210^T^ (= DSM 26640^T^) is the authentic type strain of *P. gallaeciensis*. Moreover, the genome sequenced strain ANG1 has been referred to as *P. gallaeciensis* based on 16S rRNA analyses [63], but our recent study revealed a well-supported association with *P. caeruleus* and *P. daeponensis* [4]. The relationships between these *Phaeobacter* strains have not been coroborated using genome sequences. Thus, we used the Genome-to-Genome Distance Calculator (GGDC) [64] to investigate the affiliation of strain ANG1 and the genomic similarities between *P. inhibens* and *P. gallaeciensis* strains from available genome sequences and conducted phylogenomic analyses to address the relationship between *P. gallaeciensis* and *P. inhibens*.

Table 8 shows the results of the calculated digital DNA-DNA hybridization (DDH) similarities of *P. gallaeciensis* CIP 105210^T^ and *P. inhibens* DSM 16374^T^ (T5^T^) to other *Phaeobacter* strains. For DDH values ≤70% the respective query strain would be considered as belonging to a different species than the strain used as a reference [65,66].

**Table 8:** DDH similarities with standard deviations between *P. gallaeciensis* CIP 105210^T^, *P. inhibens* DSM 16374^T^ (T5^T^) and other *Phaeobacter* strains calculated in silico with the GGDC server version 2.0 [64]. The numbers in parentheses are IMG Taxon IDs identifying the genome sequence.

Formula reference species	identities/HSP length [%] *P. gallaeciensis* DSM 26640^T^ (= CIP 105210^T^ = BS107^T^)	identities/HSP length [%] *P. inhibens* DSM 16374^T^ (T5^T^)
*P. inhibens* DSM 24588 (2.10) (2501651220)	38.00% ± 2.49	79.50% ± 2.80
*P. inhibens* DSM 17395 (2510065029)	38.40% ± 2.50	78.70% ± 2.83
*P. gallaeciensis* ANG 1 (2526164696)	21.40% ± 2.34	21.10% ± 2.33
*P. inhibens* DSM 16374^T^ (T5^T^) (2516653078)	38.20% ± 2.50	100%
*P. gallaeciensis* DSM 26640^T^ (= CIP 105210^T^) (2545555837)	100%	38.20% ± 2.50

With the exception of *P. gallaeciensis* ANG 1, which neither belongs to *P. gallaeciensis* nor *P. inhibens* based on DDH values, the analysis supports the current classification. *P. inhibens* with the type strain DSM 16374^T^ (T5^T^) includes the strains DSM 17395 and DSM 24588 (2.10), whereas the strain *P. gallaeciensis* CIP 105210^T^ (= DSM 26640^T^) is the sole representative of *P. gallaeciensis* analyzed in the current study.

For the phylogenomic analysis, protein sequences from the available *Phaeobacter* genomes were retrieved from the IMG website (*P. arcticus* DSM 23566^T^; ID 2516653081; *P. caeruleus* DSM 24564^T^ (13^T^), ID 2512047087; *P. daeponensis* DSM 23529^T^ (TF-218^T^), ID 2516493020; *P. inhibens* DSM 16374^T^ (T5^T^), ID 2516653078) or from NCBI (*P. inhibens* DSM 24588 (2.10), CP002972 – CP002975; *P. sp.* ANG1, AFCF00000000; *P. gallaeciensis* CIP 105210^T^ (= DSM 26640^T^), AOQA00000000; *P. inhibens* DSM 17395, CP002976 – CP002979; *P.* sp. Y4I, ABXF00000000).

These sequences were investigated using the DSMZ phylogenomics pipeline as previously described [67-70] using NCBI BLAST [71], TribeMCL [72], OrthoMCL [73], MUSCLE [74], RASCAL [75], GBLOCKS [68] and MARE [76] to generate gene- and ortholog-content matrices as well as concatenated alignments of distinct selections of genes.

Maximum likelihood (ML) [77] and maximum-parsimony (MP) [78,79] trees were inferred from the data matrices with RAxML [80,81] and PAUP* [82], respectively, as previously described [68,70,72,83].

**Figure 5:**
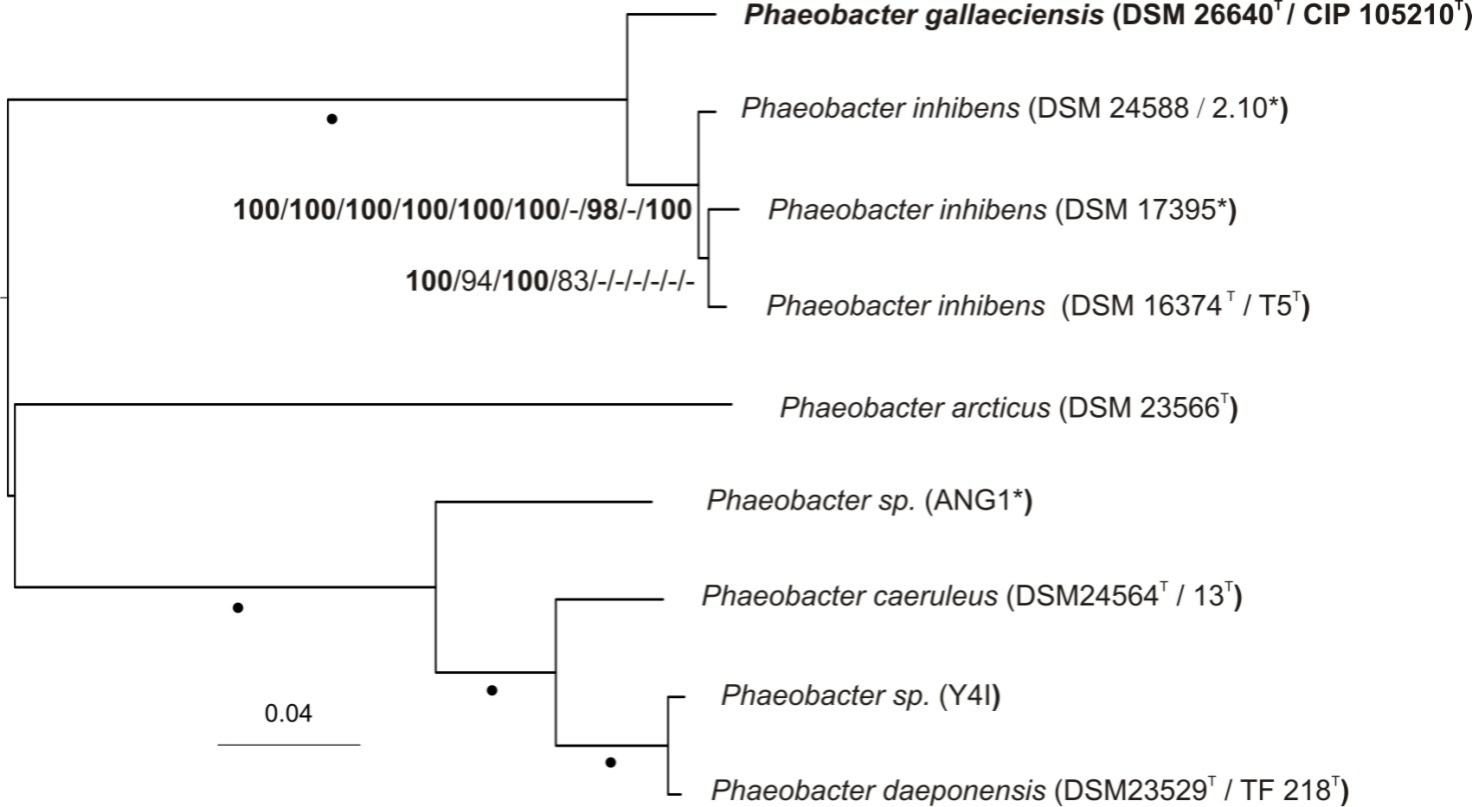
Phylogenetic tree inferred from the MARE-filtered supermatrix under the maximum likelihood (ML) criterion [77] and rooted using the midpoint rooting approach [84]. The branches are scaled in terms of the expected number of substitutions per site. Numbers above the branches (from left to right) are bootstrap support values [85] (if greater than 60%) from ML/MP MARE-filtered supermatrix; ML/MP unfiltered (full) supermatrix; ML/MP core-genes supermatrix; ML/MP gene-content matrix; ML/MP ortholog-content matrix. Values larger than 95% are shown in bold; dots indicate branches with maximum support under all settings. Genomes marked with stars have been renamed according to this study and [3].

The results of the phylogenomic analyses are shown in Figure 5. The “full” and MARE-filtered supermatrix trees were topologically identical and the tree of the latter analysis is shown in Figure 5 together with ML and MP bootstrap support values from all analyses if larger than 60%. The tree inferred from the core-gene matrix showed a distinct grouping within *Phaeobacter inhibens*, i.e. *P. inhibens* DSM 17395 as sister of the clade comprising *P. inhibens* DSM 16374^T^ (T5^T^) and *P. inhibens* DSM 24588 (2.10). The topologies of both MP and ML “full” and MARE-filtered supermatrix trees were identical, whereas the MP core-genes tree was topologically identical to the ML core-genes tree. Both gene-content and ortholog-content MP trees were topologically identical and showed *P. inhibens* DSM 16374^T^ (T5^T^) as a sister taxon of *P. inhibens* DSM 24588 (2.10) and *P. inhibens* DSM 17395. Only the ML gene-content and ortholog-content trees deviated regarding the species boundaries, showing a clade comprising *P. inhibens* DSM 16374^T^ (T5^T^) and *P. gallaeciensis* CIP 105210^T^ (= DSM 26640^T^) as well as a clade comprising *P. inhibens* DSM 24588 (2.10) and *P. inhibens* DSM 17395.

Thus, the analyses supported the earlier conclusion [3] that DSM 17395 belongs to *P. inhibens*. The analyses also confirmed that *P. “gallaeciensis”* ANG1 belongs neither to *P. gallaeciensis* nor to *P. inhibens* and might therefore represent a novel, not yet named seventh species in the genus *Phaeobacter*. Further, the analysis confirms *P. gallaeciensis* CIP 105210^T^ (= DSM 26640^T^) as the sole representative of the species *Phaeobacter gallaeciensis*.
